# Species-Specific Effects of Humic Substances and Mycorrhiza on Antioxidant Defense and Metal Stress Tolerance in *Cannabis sativa*, *Sorghum sudanense × bicolor*, and *Miscanthus × giganteus* Under Field Conditions

**DOI:** 10.3390/ijms27093942

**Published:** 2026-04-28

**Authors:** Karolina Jaros-Tsoj, Artur Nowak, Jolanta Jaroszuk-Ściseł, Piotr Sugier, Danuta Sugier, Francois Rineau, Jaco Vangronsveld, Małgorzata Wójcik

**Affiliations:** 1 Department of Plant Physiology and Biophysics, Institute of Biological Sciences, Maria Curie-Skłodowska University, 19 Akademicka Street, 20-033 Lublin, Poland; karolina.jaros-tsoj@mail.umcs.pl (K.J.-T.); jaco.vangronsveld@mail.umcs.pl (J.V.); 2 Centre for Environmental Sciences, Hasselt University, Agoralaan, Buidling D, B-3590 Diepenbeek, Belgium; francois.rineau@uhasselt.be; 3 Department of Industrial and Environmental Microbiology, Institute of Biological Sciences, Maria Curie-Skłodowska University, 19 Akademicka Street, 20-033 Lublin, Poland; artur.nowak@mail.umcs.pl (A.N.); jolanta.jaroszuk-scisel@mail.umcs.pl (J.J.-Ś.); 4 Department of Botany, Mycology and Ecology, Institute of Biological Sciences, Maria Curie-Skłodowska University, 19 Akademicka Street, 20-033 Lublin, Poland; piotr.sugier@mail.umcs.pl; 5 Department of Industrial and Medicinal Plants, University of Life Sciences in Lublin, 15 Akademicka Street, 20-950 Lublin, Poland; danuta.sugier@up.lublin.pl

**Keywords:** antioxidative enzyme activities, lipid peroxidation, proline, sugars, chronic metal exposure

## Abstract

Abiotic stresses, including heavy metal contamination, can severely impair plant growth and antioxidative defense. However, their adverse effects may be mitigated through sustainable strategies such as biostimulant application. This study investigated the effects of humic substances (HSs), alone or combined with mycorrhizal inoculation (M), on oxidative stress and antioxidative responses in *Cannabis sativa*, *Sorghum sudanense × bicolor*, and *Miscanthus × giganteus* grown under field conditions on metal-contaminated agricultural soil exceeding regulatory thresholds for Zn, Pb, and Cd. Plant growth, lipid peroxidation, stress-related metabolites (proline, sugars), antioxidative enzyme activities (catalase, CAT; ascorbate peroxidase, APX; guaiacol peroxidase, GOPX; glutathione reductase, GR, and superoxide dismutase, SOD), and leaf metal concentrations were analyzed. Biostimulants increased proline and sugars in *Sorghum* (by up to 55% and 80%, respectively), accompanied by reduced oxidative stress indicators and improved biomass (by 26%). In *Cannabis*, higher Cd and Pb concentrations following biostimulant treatments were associated with increased SOD, APX, and GR activities (by 33–267%), without affecting growth. In *Miscanthus*, increased lipid peroxidation (by 37–60%) occurred alongside enhanced GR and APX activities. These results indicate strong species-specific responses and absence of consistent synergistic effects of HSs and M, highlighting distinct physiological strategies of stress adaptation and antioxidative defense on metal-contaminated soils. Future research should address physiological and molecular mechanisms underlying these responses.

## 1. Introduction

Plants experience a wide range of abiotic and biotic stresses in their natural environments, which affect their physiology and metabolic processes, ultimately leading to reduced growth, development, and productivity. It is estimated that environmental constraints affect approximately 96.5% of the Earth’s land surface [1]. Soil contamination with heavy metals (HMs), defined as metals and metalloids with relatively high density (typically >5 g cm^−3^), commonly considered as trace elements potentially toxic to living organisms, has become a serious problem over recent decades, primarily due to anthropogenic activities, including ore mining and processing, industrialization, urbanization, transportation, and intensive agricultural practices [2]. It is estimated that 14–17% of agricultural lands exceed permissible thresholds for at least one toxic metal [3]. The HM group includes mercury (Hg), lead (Pb), cadmium (Cd), chromium (Cr), nickel (Ni), copper (Cu), and zinc (Zn). Some of these elements, such as Cu, Zn, and Ni, are essential micronutrients for plants at certain concentrations; however, their excess leads to toxic effects on plant growth and metabolism [4]. Excessive HM concentrations disrupt photosynthesis, respiration, water balance, and mineral nutrition, and adversely affect cell structure and ultrastructure [5]. HM toxicity primarily results from interactions with functional groups of macromolecules, leading to enzyme inactivation and denaturation, alterations in protein structure, and disruption of membrane integrity. In addition, excessive accumulation of toxic HMs in plant tissues induces enhanced production of reactive oxygen species (ROS), ultimately disrupting cellular redox homeostasis and causing oxidative stress [5,6,7].

ROS are by-products of aerobic metabolism resulting from the incomplete reduction of molecular oxygen, leading to the presence of at least one unpaired electron in their molecules. They are formed in cellular compartments characterized by intense electron flow, particularly mitochondria and chloroplasts, but also in peroxisomes, the endoplasmic reticulum, the plasma membrane, and the apoplast [6,8]. ROS include singlet oxygen (^1^O_2_), triplet oxygen (^3^O_2_), superoxide anion (O_2_•^−^), hydrogen peroxide (H_2_O_2_), hydroxyl radical (•OH), hydroperoxyl radical (HO_2_•), and alkoxyl radical (RO•). One of the most extensively studied ROS-induced processes is peroxidation of membrane lipids, which results in increased membrane permeability and uncontrolled leakage of electrolytes. Free radicals also induce morphological alterations in mitochondrial outer membranes, leading to loss of membrane integrity, as well as damage to chloroplast membranes, causing their swelling and rupture. Excessive ROS accumulation can damage nucleic acids by inducing nucleotide deletions, dissociation of proteins covalently bound to DNA, and single-strand DNA breaks. In addition, ROS oxidize functional groups of enzymes, resulting in enzymatic inactivation and profound alterations in cellular metabolism [9].

Despite their well-documented toxicity, ROS can also function as important signaling molecules that interact with other signaling pathways, such as calcium ion (Ca^2+^) signaling, mitogen-activated protein kinase (MAPK) cascades, nitric oxide (NO) signaling, and phytohormones, thereby modulating stress responses and plant growth [8]. They are also involved in intercellular communication through the so-called “ROS wave”, which enhances systemic stress responses in plants [10]. However, excessive ROS accumulation results in cellular damage and may ultimately lead to cell death.

To ensure normal growth and prevent oxidative stress-induced damage, plants activate an antioxidant defense system composed of enzymatic and non-enzymatic components that function synergistically to alleviate the harmful effects of excess ROS [8,11,12]. Enzymatic antioxidants include: superoxide dismutase (SOD), catalase (CAT), ascorbate peroxidase (APX), glutathione peroxidase (GPX), guaiacol peroxidase (GOPX), and glutathione reductase (GR). This enzymatic machinery is supported by several non-enzymatic antioxidants, particularly ascorbic acid (AsA), reduced glutathione (GSH), carotenoids, and flavonoids. In addition, other stress-related metabolites with antioxidative properties, such as proline and sugars, play important roles in plant responses to metal-induced oxidative stress. Proline has been shown to fulfill multiple protective functions, including acting as a potent antioxidant while maintaining cellular turgor (osmotic balance), stabilizing membranes and thereby preventing lipid peroxidation, serving as a signaling molecule, and chelating metal ions [13,14]. Similarly, sugars contribute to oxidative stress mitigation not only through direct ROS scavenging but also by stabilizing cellular membranes, serving as energy sources, and acting as signaling molecules (e.g., by activating the protective pentose phosphate pathway) [15,16,17]. Therefore, selected biochemical and physiological parameters, including lipid peroxidation, stress-related metabolites (proline and sugars), and the activity of key antioxidant enzymes, were used in this study as indicators of plant oxidative stress status and defense responses.

Under normal conditions, ROS are effectively neutralized by the antioxidant defense system; however, under stress conditions, the efficiency of this system is challenged, and the capacity of enzymatic activities as well as the levels of non-enzymatic antioxidants may become insufficient to maintain proper cellular functioning. To counteract the damage caused by excessive free radical accumulation, plant cells must enhance their antioxidative capacity. Numerous studies have reported increased activities of antioxidant enzymes and elevated concentrations of non-enzymatic antioxidants in plants exposed to abiotic stress, including metal stress [7,11,18,19]. It should be noted that the magnitude of antioxidant responses depends on plant species, developmental stage, and environmental conditions, including the duration and intensity of the stress factor [9].

In the context of agricultural production efficiency, understanding the impact of metal pollution on plant metabolism and identifying strategies to improve plant performance under metal pollution are of considerable importance [2,20,21]. One promising approach involves the application of biostimulants, defined as substances that enhance plant tolerance to biotic and abiotic stresses and improve crop yield and quality by modulating key physiological and molecular pathways [22,23]. Based on their origin, biostimulants can be classified into humic substances, protein hydrolysates and other nitrogen-containing compounds, seaweed extracts, inorganic compounds, chitosan and other polymers, as well as beneficial fungi and bacteria. Humic substances (HSs) are natural components formed during decomposition of organic matter. Their stimulating effects on plants do not result directly from nutrient supply but rather from improvements in the soil environment (including pH modification), modulation of root architecture, stimulation of carbon and nitrogen metabolism, and regulation of gene expression, including genes involved in stress defense responses [24,25]. Arbuscular mycorrhizal fungi (AMF) form symbiotic associations with the roots of more than 90% of terrestrial plant species. They improve plant mineral nutrition and soil structure and are also capable of modulating secondary metabolism. AMF can sequester heavy metals within their hyphae, thereby reducing metal uptake by host plants [26,27,28]. Recent studies have further emphasized that biostimulants may regulate plant stress responses through interconnected signaling networks involving phytohormonal regulation (including abscisic acid, auxins, gibberellins, cytokinins, jasmonic acid, and ethylene), ROS–hormone cross-talk, and plant–microbe interactions. In particular, arbuscular mycorrhizal fungi and humic substances have been shown to enhance stress adaptation not only via improved nutrient acquisition but also through modulation of these regulatory pathways, thereby contributing to improved plant resilience under abiotic stress conditions [29,30,31,32]. Despite numerous studies, the mechanisms underlying biostimulant-mediated mitigation of metal-induced oxidative stress, particularly through modulation of antioxidant defense systems, remain insufficiently understood. Most existing research has focused on single plant species, controlled experimental conditions, or individual mitigation strategies, resulting in limited insight into species-specific responses and the combined effects of biostimulants under field conditions. Although field-based studies on biostimulants have increased in recent years, most are still restricted to single-species systems, and comparative analyses across different plant functional types under identical stress conditions remain scarce [27,33,34,35,36]. Moreover, although HSs and AMF are recognized for their potential to alleviate abiotic stress, their synergistic effects on oxidative stress responses, stress-related metabolites, and antioxidant enzyme activities have rarely been evaluated across plant species differing in life-history traits and taxonomic affiliation. To the best of our knowledge, this is one of the first field-based studies to simultaneously evaluate HSs and mycorrhizal inoculation across three phylogenetically and functionally distinct plant species grown under identical heavy metal-contaminated field conditions, allowing direct comparison of species-specific physiological and biochemical responses to biostimulant application.

The main objective of this study was to assess the effects of HSs and their combined application with mycorrhizal inoculation (M) on plant responses to metal-polluted soil in three species—miscanthus (*Miscanthus × giganteus*), sorghum (*Sorghum sudanense × bicolor*), and hemp (*Cannabis sativa* L.)—under field conditions. These species were chosen as model crops for phytomanagement of metal-polluted soils due to their high biomass yield, low input requirements, and tolerance to abiotic stress, including HMs [27,33,37,38]. As non-food crops, they can be cultivated on degraded land with minimal risk of entering the food chain, while providing biomass for industrial applications. In addition, the selected species represent contrasting plant functional types, differing in taxonomic affiliation (monocotyledonous vs. dicotyledonous), life-history strategy (annual vs. perennial), and photosynthetic pathway (C3 vs. C4). This diversity enabled evaluation of whether biostimulant-mediated mitigation of oxidative stress is primarily species-specific or driven by the type of biostimulant applied. Although both HSs and AMF mitigate abiotic stress, they represent distinct categories of biostimulants and operate through partially different mechanisms—HSs mainly improving soil properties and plant metabolism, whereas AMF enhancing nutrient acquisition and plant–soil interactions—suggesting their potential complementary effects.

The specific objectives of this study were to: (i) evaluate plant growth and oxidative stress levels (lipid peroxidation) in response to HS and HS + M treatments; (ii) assess the accumulation of stress-related metabolites (proline and total sugars) and the activity of key antioxidative enzymes (CAT, APX, GOPX, GR, and SOD); (iii) quantify metal accumulation in leaves and its relationship with oxidative stress and antioxidant responses; and (iv) compare responses among species to distinguish species-specific vs. biostimulant-specific effects. We hypothesized that: (1) application of HSs improves plant growth and reduces oxidative stress by enhancing antioxidant defense and stress-related metabolites; (2) HSs combined with M provides stronger stress mitigation than HSs alone due to complementary effects; and (3) the magnitude and nature of biostimulant-induced stress alleviation are species-specific, reflecting differences in plant traits and stress response mechanisms.

## 2. Results

### 2.1. Growth Parameters

**Figure 1:**
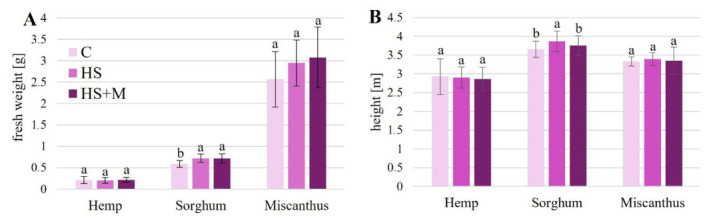
Growth parameters shoot fresh weight (**A**) and plant height (**B**) of hemp, sorghum, and miscanthus grown on HM-contaminated soil without biostimulant application (C—control) or with humic substances (HS) or humic substances combined with mycorrhiza (HS + M). In the case of miscanthus, the mean weight of a single genet is presented. Data are means ± SD, *n* = 9 (**A**) or *n* = 75 (**B**). Within each crop, means followed by the same letter do not differ significantly (*p* < 0.05).

The applied biostimulants did not significantly affect plant height or shoot fresh weight in hemp and miscanthus (Figure 1A,B). In sorghum, both biostimulant treatments resulted in an approximately 26% increase in shoot fresh weight compared to the control. However, a significant increase in plant height was observed only in response to HS application (by 5.8%), whereas HS + M had no effect on this parameter.

### 2.2. Stress-Related Metabolites

**Figure 2:**
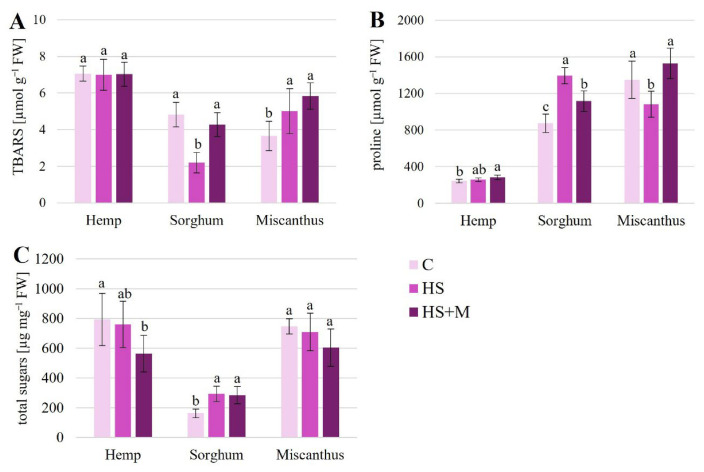
Concentrations of stress-related markers TBARS (**A**), proline (**B**), and total sugars (**C**) in leaves of hemp, sorghum, and miscanthus grown on HM-contaminated soil without biostimulant application (C—control) or with humic substances (HS) or humic substances combined with mycorrhiza (HS + M). Data are means ± SD, *n* = 9. Within each crop, means followed by the same letter do not differ significantly (*p* < 0.05).

The TBARS (thiobarbituric acid reactive substances) concentration, used as an indicator of lipid peroxidation and oxidative stress, was not significantly affected by biostimulant application in hemp leaves (Figure 2A). In sorghum, HS treatment resulted in a significant reduction in TBARS levels (by approximately 54.5%) compared to the control, whereas no such effect was observed following HS + M application (Figure 2A). In contrast, both biostimulant treatments led to a significant increase in TBARS concentration in miscanthus leaves, indicating enhanced lipid peroxidation, with increases of 37% and 60% for HS and HS + M, respectively (Figure 2A).

Proline accumulation exhibited distinct, species-specific patterns (Figure 2B). In hemp, a significant increase in proline concentration was observed only following HS + M application, with levels 16.7% higher than in control plants. In sorghum, both biostimulant treatments significantly increased proline concentration compared to the control; plants treated with HS and HS + M showed increases of 54.9% and 27.5%, respectively. Moreover, proline concentration was significantly higher in HS-treated plants than in those receiving HS + M. In contrast, in miscanthus, HS application resulted in a significant decrease in proline concentration (by 19.8%) compared to the control, whereas treatment with HS + M had no significant effect on this parameter.

Biostimulant application did not affect total carbohydrate concentrations in miscanthus leaves (Figure 2C). In hemp, HS treatment had no effect, whereas HS + M resulted in a significant decrease in total sugar concentration by approximately 28.9%. Conversely, in sorghum, both HS and HS + M treatments significantly increased total carbohydrate levels, by 80.3% and 75%, respectively, compared to the control.

### 2.3. Activity of Antioxidative Enzymes

**Figure 3:**
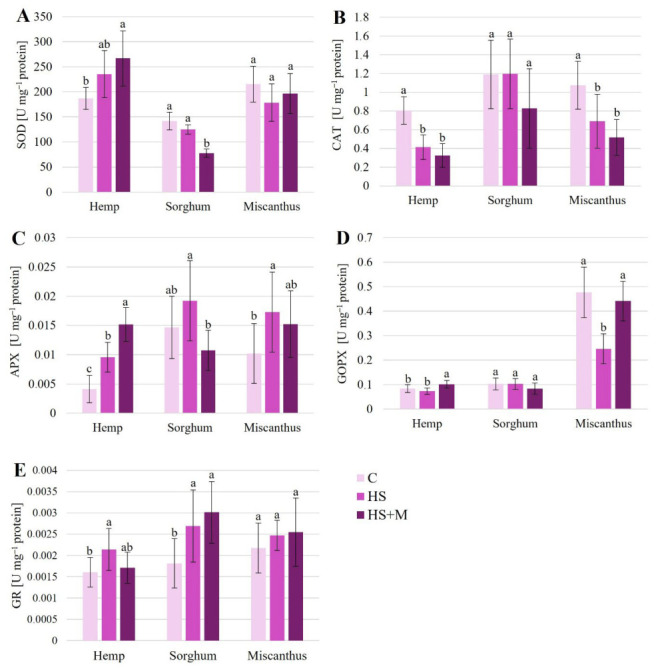
The activity of antioxidative enzymes SOD (**A**), CAT (**B**), APX (**C**), GOPX (**D**), and GR (**E**) in leaves of hemp, sorghum, and miscanthus grown on HM-contaminated soil without biostimulant application (C—control) or with humic substances (HS) or humic substances combined with mycorrhiza (HS + M). Data are means ± SD, *n* = 9. Within each crop, means followed by the same letter do not differ significantly (*p* < 0.05).

The activity of antioxidative enzymes varied among the studied species and did not follow a consistent pattern in response to biostimulant application (Figure 3).

In hemp, SOD activity was highest following HS + M treatment, reaching 42.6% above the control level. In sorghum, on the other hand, HS + M application lowered SOD activity by 45.4%, whereas in miscanthus, no significant changes in SOD activity were observed under any biostimulant treatment (Figure 3A).

Both biostimulant treatments decreased CAT activity in hemp and miscanthus (Figure 3B). In hemp, CAT activity was reduced by 48.7% and 59.5% under HS and HS + M, respectively, compared to control plants, with no significant difference between the two treatments. Similarly, in miscanthus, CAT activity decreased by 35.8% under HS and 51.8% under HS + M, without significant differences between treatments. No significant changes in CAT activity were observed in sorghum leaves.

APX activity increased by 130.9% and 266.9% in hemp following HS and HS + M application, respectively, with HS + M inducing a significantly higher increase than HS alone (Figure 3C). In sorghum, neither treatment affected APX activity. In miscanthus, HS increased APX activity by 69.8%, whereas HS + M had no significant effect.

In hemp, GOPX activity increased by 22.1% following HS + M application compared to control plants (Figure 3D). No significant changes were observed in sorghum. In miscanthus, GOPX activity decreased by 48.4% under HS treatment.

In hemp, HS increased GR activity by 33.3% relative to the control. In sorghum, GR activity enhanced by 48.2% and 65.9% under HS and HS + M, respectively. In miscanthus, no significant changes in GR activity were observed under either treatment (Figure 3E).

### 2.4. Metal Concentrations in the Leaves

**Figure 4:**
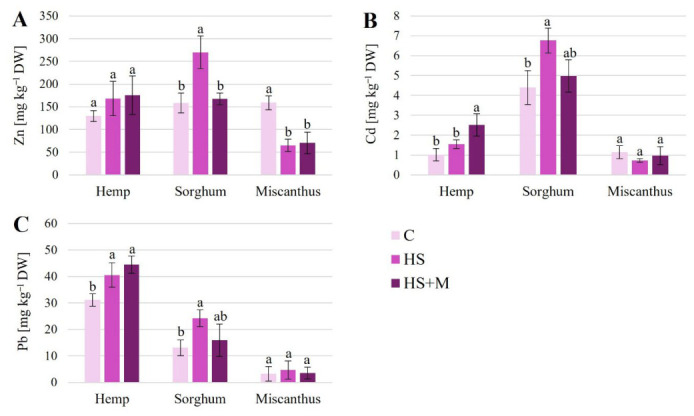
Concentrations of zinc (**A**), cadmium (**B**), and lead (**C**) in leaves of hemp, sorghum, and miscanthus grown on HM-contaminated soil without biostimulant application (C—control) or with humic substances (HS) or humic substances combined with mycorrhiza (HS + M). Data are means ± SD, *n* = 3. Within each crop, means followed by the same letter do not differ significantly (*p* < 0.05).

The concentrations of Zn, Cd, and Pb—present at elevated levels in the experimental soil—varied in the leaves in response to biostimulant application depending on the metal, treatment, and plant species (Figure 4). In hemp, a general trend of increased metal concentration in the leaf tissues was observed following HS and HS + M applications; however, statistically significant increases were only detected for Cd under HS + M treatment and for Pb under both HS and HS + M treatments. In sorghum, HS application increased Zn, Cd, and Pb concentrations in leaves compared to the control, although the differences between HS and HS + M treatments were only statistically significant for Zn. In miscanthus, leaf concentrations of Cd and Pb were not significantly affected by biostimulants, whereas both HS and HS + M treatments led to a decrease in Zn concentration. Comparing metal accumulation among the three crops investigated, sorghum exhibited the highest Cd levels, whereas hemp accumulated the highest Pb concentrations in leaf tissues. Miscanthus showed the lowest concentrations of Zn, Cd, and Pb among the species studied.

### 2.5. Comparative Overview of Biostimulant Effects in Hemp, Sorghum, and Miscanthus

**Figure 5:**
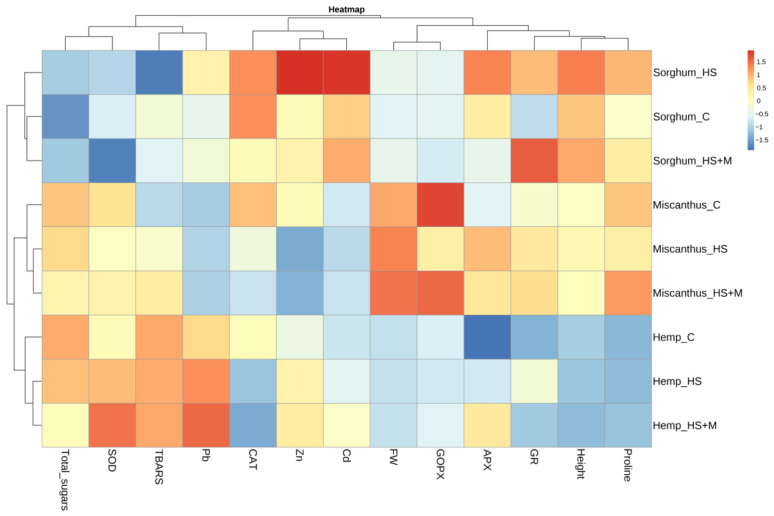
Heatmap showing standardized values of physiological, biochemical, and growth parameters in hemp, sorghum, and miscanthus grown on HM-contaminated soil without biostimulants (C, control) or with humic substances (HS) or humic substances combined with mycorrhiza (HS + M). Color scale represents relative differences in parameter values (blue—lower, red—higher). Hierarchical clustering based on Euclidean distance was applied to both variables and treatments to identify patterns of similarity. Abbreviations: FW, shoot fresh weight; TBARS, thiobarbituric acid reactive substances concentration; SOD, superoxide dismutase activity; CAT, catalase activity; APX, ascorbate peroxidase activity; GOPX, guaiacol peroxidase activity; GR, glutathione reductase activity; Cd, cadmium; Pb, lead; Zn, zinc.

The correlation heatmap revealed clear species-specific patterns in the relationships among plant growth, metal accumulation, oxidative stress markers, and physiological responses (Figure 5). Under biostimulant treatments (HS and HS + M), sorghum showed positive correlations between antioxidant enzyme activities (CAT, APX) and stress-related parameters. In miscanthus, fresh weight was positively associated with antioxidant enzyme activity, particularly GOPX. In contrast, hemp exhibited weaker or negative correlations between growth parameters and antioxidant responses, along with stronger associations between metal concentrations (especially Pb) and enzymatic activity (e.g., SOD). Overall, the observed correlation patterns highlight distinct, species-specific strategies of stress response and adaptation under field conditions, rather than a uniform effect of biostimulant application.

Furthermore, Table 1 provides a synthetic overview of the direction and statistical significance of changes in the studied parameters in response to biostimulant application across the three species, complementing the results presented in Figure 1, Figure 2, Figure 3 and Figure 4.

Across all species and parameters, the application of HS and HS + M resulted either in no effect or in significant positive and negative changes relative to the control, indicating differentiated physiological responses rather than a uniform stimulation effect. The number and direction of significant changes differed among species and between the two biostimulant treatments. In hemp, the combined treatment (HS + M) more frequently induced positive changes in stress-related parameters compared to HS alone. In contrast, the opposite trend was observed in sorghum, where HS alone often elicited stronger responses. Only two parameters—APX activity in hemp and free proline concentration in sorghum—showed a significantly greater increase under HS + M than under HS alone.

Miscanthus exhibited predominantly neutral or negative responses to biostimulant application, including increased lipid peroxidation, decreased CAT or GOPX activities, and reduced concentrations of free proline and Zn. Conversely, sorghum displayed the most pronounced positive responses, including improved growth, decreased lipid peroxidation, and increased sugar and proline concentrations, despite elevated leaf metal concentrations, particularly under HS treatment.

Given the high number of variables and the predominance of species-specific rather than treatment-dependent patterns, multivariate analysis (PCA) was applied to further explore relationships among parameters and treatments.

### 2.6. Integrated Principal Component Analysis (PCA) of Plant Responses to Biostimulant Application

**Figure 6:**
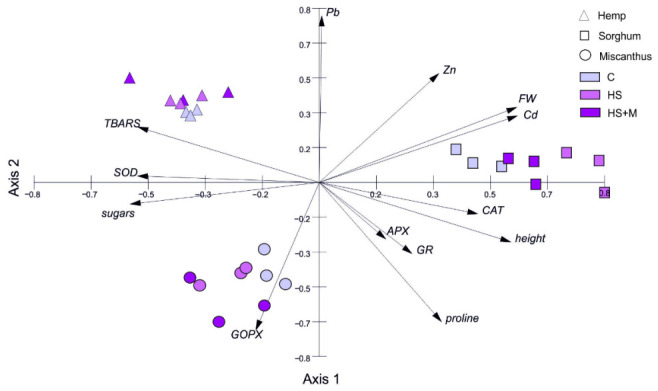
Principal component analysis (PCA) illustrating the contributions of measured variables to the responses of hemp, sorghum, and miscanthus grown on HM-contaminated soil without biostimulants (C, control) or with humic substances (HS) or humic substances combined with mycorrhiza (HS + M). For explanation of abbreviations see Figure 5.

Principal component analysis (PCA) was performed to integrate growth parameters, stress-related metabolites, antioxidative enzyme activities, and metal concentrations, and to explore overall patterns of variation among the three studied species treatments. The first two principal components (PC1 and PC2) explained 43.36% and 27.93% of the total variance, respectively, accounting for 71.29% of the overall data variability (Figure 6, Table 2). PCA confirmed strong species-specific responses to biostimulant application. The first principal component (Axis 1) clearly separated sorghum, positioned on the right side of the ordination space, from hemp on the left. PC1 was positively correlated with plant height, fresh weight, CAT activity, as well as Cd and Zn concentrations, reflecting characteristics of sorghum, while negatively correlated with TBARS concentration, SOD activity, and total sugar concentration, which were characteristic of hemp. The second principal component (Axis 2) was primarily associated with shoot Pb concentrations and negatively correlated with GOPX activity in leaf tissues. Along PC2, miscanthus was separated in the lower part of the ordination space, reflecting its distinct growth and biochemical profile relative to hemp and sorghum.

**Figure 7:**
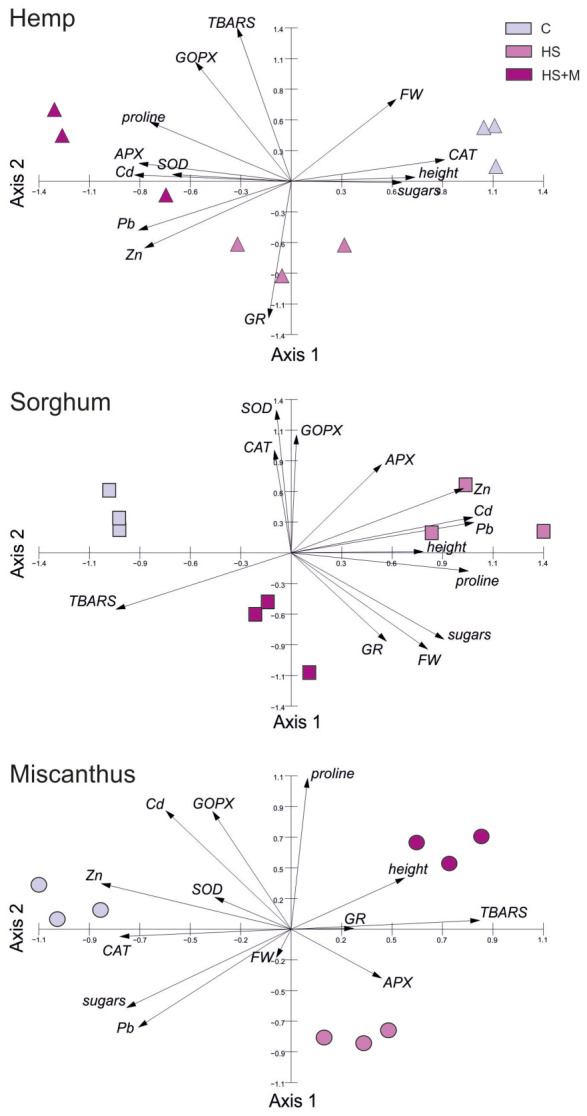
Principal component analysis (PCA) illustrating the contribution of measured variables to the individual responses of hemp, sorghum, and miscanthus grown on HM-contaminated soil without biostimulants (C, control) or with humic substances (HS) or humic substances combined with mycorrhiza (HS + M). For explanation of abbreviations see Figure 5.

The integrated PCA revealed that species-specific traits were the primary drivers of variability in the dataset. Therefore, to further explore potential trends associated with biostimulant application, additional PCA was performed separately for each species, with particular attention to parameters related to the antioxidant system (Figure 7, Table 3).

The first two principal components explained 78.54%, 71.97%, and 63.00% of the total variances for hemp, sorghum, and miscanthus, respectively, allowing reliable interpretation of the effects of biostimulant treatments on the measured parameters. In all three species, the treatment groups (control, HS, and HS + M) were clearly separated in the PCA space; however, they were associated with distinct metabolic and growth-related traits.

In hemp, the first principal component (PC1) was positively correlated with CAT activity, soluble sugar content, and plant height and fresh weight, grouping control samples on the right side of the score plot. In contrast, HS + M-treated plants were located on the left side of the diagram and were associated with higher APX and SOD activities, increased proline content, and elevated concentrations of all three metals. The second axis (PC2) was positively correlated with TBARS concentration and GOPX activity, and negatively correlated with GR activity. This axis allowed discrimination of HS-treated samples, which were located in the lower part of the plot and characterized by higher GR activity, lower GOPX activity, and reduced TBARS levels.

In sorghum, PC1 was positively correlated with plant height, proline and sugar concentrations, as well as Cd, Pb, and Zn concentrations, reflecting characteristics of the HS treatment. In contrast, PC1 was negatively correlated with TBARS concentrations, which distinguished the control samples. PC2 was positively correlated with the activities of antioxidant enzymes, including CAT, SOD, GOPX, and APX. This axis allowed discrimination of HS-treated plants, which were located in the lower part of the ordination space and were characterized by relatively low activities of these enzymes.

In miscanthus, a different pattern of sample distribution was observed. PC1 was positively correlated with TBARS concentration, APX activity, and plant height, which characterized the HS + M treatment, and negatively correlated with CAT and SOD activities as well as soluble sugar content and Cd, Pb, and Zn concentrations, distinguishing the control samples. PC2 was positively correlated with proline content and GOPX activity and allowed separation of HS-treated plants, which were positioned at negative PC2 values and showed an association with APX activity.

## 3. Discussion

Although Cd, Pb, and Zn are classified as redox-inactive metals and do not directly participate in classical Fenton or Haber–Weiss reactions, they are nevertheless potent inducers of ROS in plants via multiple indirect mechanisms. These include disruption of electron transport chains in chloroplasts, mitochondria, and peroxisomes, as well as stimulation of plasma membrane-bound NADPH oxidases [8,11,12,39]. Moreover, exposure to these metals may impair enzymatic antioxidant systems due to the strong affinity of Cd and Pb for thiol groups, and the depletion of glutathione pools, further exacerbating ROS accumulation and reducing the intrinsic capacity of plants to maintain redox homeostasis [5,39]. The present study demonstrates that biostimulant treatments significantly modulated key components of the oxidative stress responses and growth performance of industrial hemp, sorghum, and miscanthus cultivated under realistic field conditions on soil polluted with elevated concentrations of Zn, Pb, and Cd. Importantly, these effects were strongly species-specific.

### 3.1. Effect of Biostimulants on Metal Accumulation and Plant Growth

Metal accumulation in plants cultivated on polluted soils depends on multiple interacting factors, including metal speciation, plant-related traits (species, genotype, growth stage), and soil characteristics, such as total metal concentration, pH, texture, or organic matter content, which collectively determine metal mobility and phytoavailability [4,40]. Zinc, although an essential micronutrient for plants, becomes phytotoxic at elevated concentrations. Optimal tissue concentrations typically range from 25 to 150 mg kg^−1^ DW, whereas concentrations exceeding approximately 100–700 mg kg^−1^ DW may induce toxicity symptoms, depending on species sensitivity [41,42]. Cadmium and lead are non-essential elements, and their toxicity symptoms have been reported at concentrations of approximately 5–30 mg kg^−1^ DW for Cd and 30–300 mg kg^−1^ DW for Pb, depending on the plant species [4].

Although soil metal concentrations in our study clearly exceeded permissible agricultural limits [43], leaf metal concentrations remained below these toxicity thresholds, ranging approximately from 60–270 mg kg^−1^ DW for Zn, 1–7 mg kg^−1^ DW for Cd, and 4–45 mg kg^−1^ DW for Pb (Figure 4). Unexpectedly, application of HSs increased Pb concentrations in hemp leaves, as well as the concentrations of all three metals (Zn, Cd, and Pb) in sorghum (Figure 4 and Figure 7, Table 1). The combined HS + M treatment further enhanced Cd and Pb concentrations in hemp foliage. These findings contrast with our previous study [33], in which no treatment-related differences in leaf mineral profile were detected when sampling was performed a week earlier than in this study. Similarly, Ofori-Agyemang et al. [27,34] found no significant changes in shoot metal concentrations of hemp and sorghum, following application of the same biostimulants to plants grown on metal-polluted soil in France.

In contrast to hemp and sorghum, application of both HS and HS + M reduced Zn concentrations in miscanthus leaves (Figure 4 and Figure 7, Table 1). Moreover, miscanthus accumulated lower concentrations of all three metals compared to the two other species (Figure 4). These differences may reflect species-specific life strategies. Miscanthus belongs to perennial grasses, which are in general characterized as metal excluders. As a clonal species producing multiple tillers (ramets) per plant (genet), it may exhibit a biomass dilution effect, whereby absorbed metals are distributed across a larger shoot system.

Contrary to expectations, neither the commonly described mycorrhiza-associated dilution effect—attributed to metal sequestration in fungal hyphae [44]—nor the presumed immobilizing effect of HSs was consistently observed. Although HSs are widely reported as stabilizing agents that reduce HM phytoavailability through complex formation, their behavior is strongly soil dependent. Some reports noted the inhibitory effects of HSs in acidic soils while they stimulated metal availability in alkaline soils, which was explained by the contrasting properties of fulvic and humic acids that immobilize or mobilize HMs, respectively, depending on the soil pH [45].

Most studies addressing HM toxicity were conducted under controlled laboratory conditions, typically in hydroponic systems in which plants are exposed to a single metal inducing acute stress. In contrast, the effects of chronic exposure under natural conditions in multimetal-polluted soils remain much less explored. Chronic exposure to excess Zn, Cd or Pb commonly leads to leaf chlorosis and reduced stem elongation, while at the physiological level chlorophyll degradation, membrane lipid peroxidation, and disruption of the antioxidant system collapse are among the most frequently reported effects [7,18,46].

In our study, no visible toxicity symptoms such as chlorosis or stunted growth were observed. Nevertheless, we expected that the application of biostimulants would enhance plant growth, as such treatments were often reported to promote plant performance under both stressful and non-stressful environmental conditions [25,26,27,35,47,48]. These effects are commonly attributed to improved nutrient uptake and ion homeostasis, enhanced photosynthesis, modifications of phytohormone status (including increased synthesis of auxin, cytokinins, and gibberellins, along with reduced levels of abscisic acid and ethylene), modulation of reactive oxygen species metabolism, stimulation of antioxidant defense systems, as well as activation of ROS- and hormone-dependent signaling pathways (e.g., OsIAA11), as widely reported for HSs and AMF in recent studies [29,30,31,32,49,50]. Humic substances applied alone increased plant fresh weight and height in sorghum and also increased sorghum fresh weight when combined with mycorrhiza. In hemp and miscanthus, however, biostimulant applications did not affect growth parameters (Figure 1, Table 1). This lack of response, particularly in the case of combined HS + M treatment, suggests that the effects of biostimulants are not necessarily additive and may depend on species-specific physiological traits and environmental conditions.

Our previous study similarly showed a positive effect of HS, but not HS + M, on shoot fresh weight in sorghum and hemp [33]. Likewise, a parallel experiment conducted on metal-polluted soil in France found no increase in biomass in hemp and sorghum following treatment with the same biostimulants [27,34]. Other studies reported contrasting results, with some demonstrating positive effects of HS or HS + M on plant biomass on metal-contaminated soils [51,52,53], while others reported no significant impact [54,55].

The effects of HSs on plant growth may vary depending on their concentration, source, and—as also indicated in our study—plant species [56]. The effectiveness of AMF largely depends on the degree of root colonization, which is influenced by soil fertility and metal toxicity [53]. Colonization rates are generally higher in unpolluted than in polluted soils, and tend to be greatest in loamy soils [44]. Our experiment was conducted in a fertile loamy soil containing elevated levels of Cd, Pb, and Zn [33], which may have limited AMF root colonization. However, root colonization was not directly assessed in this study, thereby limiting a definitive interpretation of the observed responses. It should be noted that under field conditions, responses attributed to arbuscular mycorrhizal inoculation may also result from interactions between the introduced inoculum and the native soil microbiota [26,29,44,48]. Therefore, plant responses should be interpreted as the combined outcome of inoculated fungi and indigenous microbial communities naturally present in the soil. This aspect is currently being analyzed.

It has also been reported that AMF exert different effects on C4 and C3 plants, with colonization rates typically being higher in C3 species than in C4 species, which may translate, among other effects, into higher nutritional content in C3 plants as compared to C4 plants [44]. However, such a relationship was not confirmed in our study.

Overall, the contrasting responses observed among sorghum, hemp, and miscanthus highlight a strong species-specific component in plant responses to biostimulant application under multimetal soil conditions, likely reflecting differences in intrinsic stress tolerance strategies, metabolic regulation, and interactions with the soil microbiome. These results further suggest that under chronic field exposure to multiple metals, plant responses to biostimulants may be limited, possibly reflecting plant acclimation to long-term stress as well as environmental constraints affecting AMF performance.

### 3.2. Effect of Biostimulants on Stress Metabolites and Antioxidant System

**Figure 8:**
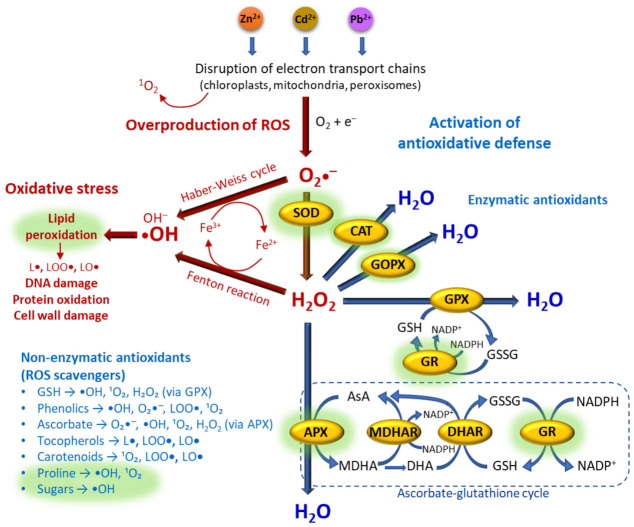
Reactive oxygen species (ROS) production and scavenging in plant cells following exposure to Zn, Cd, and Pb ions. The superoxide anion (O_2_•^−^), resulting, among others, from disruption of electron transport chains, is converted into hydrogen peroxide (H_2_O_2_) by superoxide dismutase (SOD). The H_2_O_2_ is subsequently detoxified to water (H_2_O) either by catalase (CAT), guaiacol peroxidase (GOPX), glutathione peroxidase (GPX), or ascorbate peroxidase (APX). The ascorbate–glutathione cycle also contributes to H_2_O_2_ neutralization and involves enzymes such as monodehydroascorbate reductase (MDHAR), dehydroascorbate reductase (DHAR), and glutathione reductase (GR), as well as metabolites including ascorbate (AsA), monodehydroascorbate (MDHA), dehydroascorbate (DHA), glutathione (GSH), and reduced nicotinamide adenine dinucleotide phosphate (NADPH), with the oxidized forms of the latter two being glutathione disulfide (GSSG) and nicotinamide adenine dinucleotide phosphate (NADP^+^), respectively. The hydroxyl radical (•OH) is generated via the Fenton reaction or the Haber–Weiss cycle in the presence of Fe ions and induces oxidative damage to lipids, proteins and nucleic acids, being oxidative stress symptoms. Non-enzymatic antioxidants contribute to ROS detoxification by scavenging •OH, O_2_•^−^, H_2_O_2_, and singlet oxygen (^1^O_2_), as well as lipid peroxidation products, including lipid alkyl (L•), lipid peroxyl (LOO•), and lipid alkoxyl (LO•) radicals. Metabolites highlighted in green indicate compounds analyzed in this study.

Excessive accumulation of HMs, such as Zn, Cd, and Pb, disrupts ROS homeostasis, ultimately leading to oxidative stress in plant cells [39,46,57,58]. Among ROS, the superoxide anion (O_2_•^−^) is typically the primary species generated under physiological conditions. Although relatively unreactive towards most cellular molecules, it serves as a precursor for the formation of other ROS [59]. Superoxide can be dismutated into H_2_O_2_ by SOD or converted into •OH via the Haber Weiss cycle. Hydroxyl radicals can also be generated from H_2_O_2_ in the Fenton reaction, in which the O-O double bond in this molecule is cleaved [8]. Hydroxyl radicals are highly reactive and can interact with a range of biological molecules, oxidizing cell wall polysaccharides, proteins and membrane lipids, which may lead to the formation of secondary radicals such as lipid, protein and nucleic acid peroxides. To prevent such peroxidative chain reactions and the resulting damage to cellular structure, ultrastructure, and metabolism, plants rely on a coordinated antioxidant defense system composed of enzymatic and non-enzymatic components that eliminate hydroxyl radicals and their precursors. A schematic overview of ROS generation and the antioxidant defense machinery is presented in Figure 8, with the components investigated in this study highlighted. Changes in ROS-related metabolites and antioxidant enzyme activities can therefore provide valuable insight into the physiological mechanisms underlying plant responses to heavy metal exposure and biostimulant application. In addition to redox regulation, plant responses to oxidative stress are known to be tightly controlled by hormone-mediated signaling pathways, particularly those involving abscisic acid, auxin, and ethylene, which integrate environmental cues with growth and defense responses. Although these pathways were not directly investigated in the present study, previous reports indicate that biostimulants such as HSs and AMF may modulate hormonal signaling, thereby influencing stress responses [25,30,31,32,50], and represent an important direction for future research.

The responses of hemp, sorghum, and miscanthus to metal-induced oxidative stress were markedly species-specific. Although environmental stress is generally expected to increase ROS production—thereby promoting lipid peroxidation (commonly measured as TBARS) and stimulating both enzymatic (mainly SOD, CAT, and APX, but also GR and GOPX) and non-enzymatic (e.g., proline, sugars) components of the antioxidant system—this classical linear model of oxidative stress is rarely observed in practice, reflecting the dynamic and tightly regulated nature of plant stress responses under complex environmental conditions. Indeed, numerous studies have reported considerable interspecific variation in antioxidant defense responses among plants exposed to HMs. For example, Hachani et al. [46] examined four Fabaceae species grown in a pot experiment on multimetal-polluted soil and found that lipid peroxidation was significantly elevated only in *Lens culinaris* Medik. and *Medicago arborea* L., compared with plants cultivated on uncontaminated reference soil. At the same time, these two species showed the highest increases in proline concentration. *M. arborea* exhibited a significant increase in APX and GOPX activities but not CAT activity, whereas *Vicia faba* L. revealed enhanced CAT activity while APX and GOPX remained unaffected. In *Matricaria chamomilla* L. grown in artificially Cd- or Pb-contaminated soils in a pot experiment, metal stress significantly increased lipid peroxidation, proline accumulation, and SOD activity, whereas CAT activity remained unchanged [60]. Similarly, Cd exposure increased POD and SOD activities in garden cress (*Lepidium sativum* L.) [45].

In our study, among the three crops chronically exposed to HMs under field conditions, the highest level of lipid peroxidation was found in hemp (Figure 2 and Figure 6). At the same time, hemp exhibited the lowest proline concentrations (Figure 2) and relatively low activities of APX, GOPX, and CAT (Figure 3). In contrast, miscanthus showed the lowest TBARS levels, together with the highest concentrations of non-enzymatic antioxidants (Figure 2) and the highest GOPX activity (Figure 3). These contrasting patterns suggest the operation of distinct stress mitigation strategies, in which different species rely to varying extents on enzymatic versus non-enzymatic antioxidant components. Such differences may also reflect species-specific regulation of redox homeostasis and metabolic flexibility under long-term metal exposure.

Numerous studies have demonstrated that application of biostimulants, including HSs and AMF, can alleviate metal stress and other abiotic stresses associated with oxidative damage in plant cells. These effects are typically manifested by reduced membrane lipid peroxidation (TBARS), increased levels of beneficial stress-related metabolites with osmoregulatory and antioxidative potential, including free proline and soluble sugars, and enhanced activities of antioxidant enzymes [25,30,44,47,48,61]. For instance, application of HSs (humic acids and fulvic acids) to a Cd-polluted soil decreased H_2_O_2_ and TBARS levels and increased peroxidase activity in garden cress [45]. Interestingly, proline and sucrose concentrations, as well as CAT and SOD activities, were also reduced compared with Cd-exposed plants. A similar effect was observed in Cd-exposed lettuce (*Lactuca sativa* L.), where treatment with fulvic acids lowered lipid peroxidation and enhanced ROS-scavenging capacity by increasing APX and CAT activities [62]. Humic substances were also found to enhance antioxidative enzyme activities in Cd-exposed rice (*Oryza sativa* L.) [63], and to reduce lipid peroxidation and proline concentration in Cd-treated strawberry (*Fragaria × ananassa* Duch.) [64]. Decreased APX and CAT activities as well as H_2_O_2_ and malondialdehyde (MDA) levels were reported in rapeseed (*Brassica napus* L.) grown on metal-polluted soil [65].

In pot experiments, Rasouli et al. [66] demonstrated that AMF inoculation of *Lavandula angustifolia* L. grown on Pb- and Ni-polluted soils increased metal tolerance, as reflected by improved plant growth. Interestingly, this was accompanied by increased metal accumulation and lipid peroxidation but also by enhanced activities of SOD, GOPX, and APX, together with higher proline content. Similarly, inoculation of pea (*Pisum sativum* L.) with *Glomus mosseae* improved plant performance in Pb-, Cd-, and As-polluted soil, likely through increased activities of antioxidant enzymes (CAT, APX, SOD), whereas proline decreased [67]. Furthermore, experiments with AMF inoculated sunflower (*Helianthus annuus* L.) cultivated on metal-polluted soil under field conditions showed lower lipid peroxidation and lower SOD and GOPX activities, along with decreased leaf metal concentrations, indicating a positive effect of mycorrhizal colonization [36]. Taken together, the available evidence indicates that the effects of HSs and AMF on oxidative stress responses are highly species-specific and depend on both environmental conditions and the physiological status of the plant.

Our results, in line with the results presented above, did not reveal a uniform pattern of biostimulant effects on the antioxidant defense system across the three studied species. Moreover, to the best of our knowledge, this is the first report describing the concomitant effect of HSs and AMF on the functioning of the antioxidant system in plants exposed to metal stress. The most evident observation is lack of a biostimulant-specific effect, and that simultaneous application of HS + M did not result in a synergistic enhancement of antioxidant pathways or stress metabolite accumulation (with two exceptions) (Table 1, Figure 6 and Figure 7). This lack of synergistic interaction suggests that HSs and AMF may act through partially overlapping or context-dependent mechanisms, and that their combined effects are not necessarily additive, particularly under complex field conditions. Instead, a clearly species-specific response to biostimulant application was observed.

Sorghum was the only species that exhibited increased height and fresh weight following HS treatment and increased fresh weight following HS + M treatment, despite elevated foliar Zn, Cd, and Pb concentrations in HS-treated plants. This response may be associated with a strong non-enzymatic antioxidant system, reflected by significantly increased concentrations of proline and sugars, which may compensate for the lack of induction of antioxidant enzymes by HSs or even the decreased SOD and APX activities under HS + M treatment. Such a response may reflect a shift toward metabolite-based stress mitigation, where osmolytes such as proline and soluble sugars contribute to cellular protection, osmotic adjustment, and maintenance of redox balance under metal stress. Moreover, HSs reduced the lipid peroxidation level in comparison to plants grown without biostimulant application. These findings are consistent with our previous study, where accumulation of proline was also stimulated by biostimulants in sorghum [33]. Additionally, sorghum differed from hemp by exhibiting higher chlorophyll concentrations, higher CO_2_ assimilation rates, and a higher water use efficiency (WUE) index, in response to biostimulant application. This may be related to the life strategy of sorghum as an annual C4 plant. In general, C4 plants exhibit greater tolerance to environmental stresses and lower, more controlled levels of oxidative damage compared with C3 plants (e.g., hemp), largely due to reduced photorespiration and a more efficient antioxidant system [68]. Indeed, the CO_2_-concentrating mechanism operating in the bundle sheath cells of C4 plants limits the oxygenase activity of Rubisco and reduces photorespiration, which eventually leads not only to higher photosynthesis rates but also to lower ROS production, particularly H_2_O_2_, compared with C3 plants [69,70,71]. Moreover, due to these metabolic characteristics, C4 plants are generally more resilient to drought and heat stress, which are increasingly pronounced under field conditions due to global climate change [71]. It cannot be excluded that these additional environmental stressors also affected the crops in our experiment alongside HM stress.

In contrast to sorghum, hemp growth was not influenced by biostimulant application, nor was the level of lipid peroxidation, although it remained consistently higher across all experimental variants (Figure 2A and Figure 6). Nevertheless, biostimulant application significantly enhanced the activities of several antioxidant enzymes, including APX and GR under HS treatments and SOD, APX, and GOPX activities under HS + M treatment, while CAT activity was significantly lower in both treatments. The enhanced enzymatic activity may at least partially compensate the potential deleterious effects associated with increased Cd and Pb concentrations under HS + M and Pb concentration under HS treatment (Table 1). Non-enzymatic antioxidants appeared to play a less prominent role in hemp, although free proline levels increased following HS + M application. PCA further confirmed, that biostimulant application, particularly HS + M, was correlated with increased activities of APX, SOD, GOPX, and GR, together with higher proline concentrations and elevated metal accumulations in leaf tissues (Figure 7). These results suggest that the protective role of biostimulants in alleviating metal toxicity in hemp may primarily rely on stimulation of the enzymatic antioxidant defense system. This strategy may indicate a reliance on inducible enzymatic defenses rather than constitutive metabolic protection.

Miscanthus was the least responsive species to biostimulant application among the three crops studied and, in some cases, even showed a negative response in terms of antioxidant system performance, more pronounced under HS than HS + M treatment (Table 1). This was reflected by increased TBARS production, lower proline concentrations and CAT and GOPX activities. Also, the Zn concentrations in the shoots were lower compared with plants grown without biostimulant treatment. These results could suggest enhanced oxidative stress in miscanthus plants treated with biostimulants, although this did not translate into any negative effects on plant growth (Figure 1). Such a response may reflect either increased sensitivity to biostimulant-induced metabolic shifts or a transient oxidative imbalance associated with physiological adjustment processes rather than irreversible cellular damage. The interpretation of these observations remains challenging, especially considering that miscanthus is a perennial C4 grass and that the plantation was already well established in its second growth season.

Some studies indicate that perennial grass species may exhibit greater resistance to environmental stresses such as drought or high temperature compared with annual species, displaying less pronounced leaf senescence and lower lipid peroxidation due to more developed root systems, longer growing seasons, and ability to store resources across multiple years [72,73]. However, these comparisons were typically made between closely related turfgrass species with similar ecological strategies, such as annual and perennial ryegrass and bluegrass species [72], and therefore cannot be directly extrapolated to high-biomass lignocellulosic crops such as sorghum and miscanthus. These two C4 grasses differ not only in life cycle (annual *vs.* perennial) but also growth strategies and specific adaptations to environmental challenges. Sorghum is highly sensitive to low temperatures, particularly during germination and early growth stages, but shows high drought resistance and specialized physiological mechanisms enabling survival under arid conditions. In contrast, miscanthus is better adapted to temperate climates, demonstrates superior chilling tolerance, and maintains high water-use efficiency [74]. Testa et al. [75], who compared five lignocellulosic Poaceae species in a two-year pot experiment using metal-polluted soil, reported that sorghum generally exhibited higher tolerance to certain heavy metals such as Cd and Pb compared with miscanthus at low to moderate contamination levels, whereas miscanthus showed greater tolerance to higher Zn concentrations when assessed based on growth parameters. However, the physiological mechanisms underlying these differences should still be elucidated.

Nadgórska-Socha et al. [18] and Păun et al. [36] suggested that changes in antioxidative enzyme activities observed in field studies conducted on moderately polluted soils may serve as useful biomarkers in studies related to revegetation of heavy metal-polluted sites. However, our studies clearly demonstrate that oxidative stress-related parameters are highly species-specific and, therefore, cannot be considered reliable universal indicators of environmental stress or robust predictors of species suitability for management of such marginal lands. Overall, these findings highlight the existence of distinct physiological strategies for coping with metal-induced oxidative stress among the studied bioenergy crops, which may determine their suitability for cultivation on metal-contaminated soils.

It should be noted that the lack of molecular-level analyses limits a more detailed understanding of the regulatory mechanisms underlying the observed physiological responses. In particular, gene expression profiling and direct assessment of signaling pathways would provide valuable insight into the regulation of antioxidant systems and plant–microbe interactions under multimetal stress conditions.

## 4. Materials and Methods

### 4.1. Experimental Design and Plant Material

The experimental field was located in Piekary Śląskie (50°21′19″ N; 19°00′17″ E), southern Poland, within the Upper Silesian Industrial Region. The soil properties and climatic conditions of the site were described previously [33]. The soil is a silty loam with a slightly alkaline pH (H_2_O 7.37–7.60; KCl 6.59–6.75), which may influence the mobility and bioavailability of metal(loid)s. The soil concentrations of Zn, Pb, and Cd significantly exceeded the permissible threshold values for agricultural soils established by the Polish Ministry of Climate and Environment [43], which are 1000, 500, and 5 mg kg^−1^, respectively. The average total concentrations of these elements were 8057 mg kg^−1^ Zn, 2940 mg kg^−1^ Pb, 51.56 mg kg^−1^ Cd, and 94.13 mg kg^−1^ As [33]. However, arsenic was not further evaluated in this study due to its negligible accumulation in plant tissues, remaining at trace levels substantially below reported phytotoxic ranges [4,33]. A field experiment was conducted throughout the 2023 growing season.

Three plant species were investigated in this study: miscanthus (*Miscanthus × giganteus*), industrial hemp (*Cannabis sativa* L. var. Futura 75), and sorghum (*Sorghum sudanense × bicolor* var. Bulldozer). Five voucher specimens were deposited in the Botanical Collection of the Institute of Biological Sciences of Maria Curie-Skłodowska University in Lublin (reference material No. LBL P-100001–100005). Miscanthus is a perennial monocotyledonous grass (family Poaceae) with a C4 photosynthetic pathway; its plantation was established from rhizomes in the year preceding the analyses (2022). In contrast, hemp and sorghum are annual crops established from seeds sown in May 2023. Hemp is a dicotyledonous species (family Cannabaceae) characterized by a C3 photosynthetic pathway, whereas sorghum is a monocotyledonous grass (family Poaceae) with C4 photosynthesis.

Each crop was cultivated in 8 m × 8 m plots arranged in three replicates per treatment. Within each plot, the distance between rows was 50 cm, and the distance between plants within each row was 10 cm for hemp or 15 cm for sorghum. For miscanthus, the distance between rows was 70 cm, and the distance between plants within each row was also 70 cm. The plots were hand-weeded twice (June, July) during the growing season, and no herbicides or pesticides were applied throughout the experiment.

Three treatments were implemented in triplicate for each crop: (i) control (C), without any biostimulant; (ii) humic substances (HSs); (iii) HSs combined with mycorrhiza (HS + M). Commercially available biostimulants, humic substances (Lonite, Alba Milagro, Parabiago, Italy) and mycorrhizal inoculum (Symbivit^®^, Symbiom, Lanškroun, Czech Republic) were applied according to the manufacturers’ protocols. The mycorrhizal inoculum was applied to the soil at the time of sowing of hemp and sorghum at a rate of 20 g per linear meter, placed at the bottom of the planting furrow. For miscanthus, 10 g of inoculum was applied per planting hole, directly beneath the rhizomes during plantation establishment. Humic substances were applied via root irrigation twice: the first when hemp and sorghum plants reached the 4–6 leaf stage, and again four weeks later. Miscanthus plants were treated with HSs at the same time and in the same manner as the other crops. It should be noted that under field conditions, the soil is not sterile and contains native microbial communities, including naturally occurring mycorrhizal fungi. Therefore, it is not possible to completely exclude interactions between the applied mycorrhizal inoculum and indigenous soil microorganisms. However, all treatments were established under the same field conditions and arranged in a randomized block design, which allowed for reliable comparison of treatment effects.

### 4.2. Assessing Plant Growth and Preparing Leaf Samples for Analyses

Shoot fresh weight and total plant height were determined at the beginning of September 2023 using at least 25 plants randomly collected from each experimental plot. For fresh weight assessment, three groups of 25 plants per plot were weighed, and the mean fresh weight per plant was calculated. At the same time, leaf samples were collected for the analysis of selected metabolites (total sugars, free proline, and lipid peroxidation products) and antioxidant enzyme activities, as well as for mineral determination. Samples (0.2 g fresh weight, FW) were frozen in liquid nitrogen and stored at −80 °C until biochemical analyses. Samples of the same leaves were thoroughly washed with tap water, rinsed with distilled water, and dried at 105 °C to constant weight to determine dry weight (DW) and elemental concentrations.

### 4.3. Determination of Lipid Peroxidation, Proline and Sugar Concentrations

The level of lipid peroxidation was determined based on the concentration of thiobarbituric acid reactive substances (TBARS), as described previously [29,70]. Briefly, leaf tissue samples were homogenized in 0.1% (*w*/*v*) trichloroacetic acid (TCA), centrifuged, and the resulting supernatant was mixed with 0.5% 2-thiobarbituric acid (TBA) dissolved in 20% TCA. The mixture was incubated at 95 °C for 30 min, cooled on ice, and centrifuged again. Absorbance was measured at 532 nm and corrected for nonspecific absorbance at 600 nm. The TBARS content was calculated using an extinction coefficient of 155 mM^−1^ cm^−1^.

The concentration of free proline was quantified colorimetrically using the ninhydrin reaction according to previously described protocols [33,76]. The resulting chromophore was measured spectrophotometrically at 520 nm. Proline concentrations were calculated using a standard curve and expressed in μmol g^−1^ FW.

Total carbohydrate concentrations in leaf tissues were determined using the phenol-sulfuric acid colorimetric method, as described previously [33]. Absorbance was measured at 490 nm, and carbohydrate concentrations were calculated based on a calibration curve and expressed as μg g^−1^ FW.

### 4.4. Determination of Antioxidant Enzyme Activity

Antioxidant enzyme activities were determined according to the method described by García-Limones et al. [77], as modified by Nowak et al. [78]. Briefly, 0.2 g of frozen leaf tissue was homogenized for 1 min in 6 mL of extraction buffer containing 1 mM ethylenediaminetetraacetic acid (EDTA), 1 mM phenylmethylsulfonyl fluoride (PMSF), and 1% (*w*/*v*) polyvinylpolypyrrolidone (PVPP). The resulting homogenate was filtered through Miracloth (Merck Millipore, Burlington, MA, USA) and centrifuged for 15 min at 10,000 rpm (g = 10,733) at 4 °C (MPW 352-R, Warsaw, Poland). All steps were performed on ice. Protein concentration in the resulting extracts was determined by the Bradford method [79]. The obtained extracts were subsequently used for determination of antioxidant enzyme activities.

#### 4.4.1. Superoxide Dismutase (SOD, EC 1.15.1.1) Activity

The reaction mixture in a multiwell plate consisted of extract (10, 15, or 20 µL) and phosphate buffer (50 mM, pH 7.8; 190, 185, or 180 µL, respectively), followed by 200 µL of 500 µM EDTA, 200 µL of 65 mM methionine, and 200 µL of 225 µM NBT. The reaction was initiated by adding 200 µL of 10 µM riboflavin. The reaction mixture was exposed to continuous illumination for 12 min. Absorbance was measured at 560 nm. One unit of SOD activity was defined as the amount of enzyme causing 50% inhibition of the photochemical reduction of NBT and expressed as U mg^−1^ protein.

#### 4.4.2. Catalase (CAT, EC 1.11.1.6) Activity

The reaction mixture consisted of 100 µL of extract and 400 µL of phosphate buffer (100 mM, pH 6.5). The reaction was initiated by the addition of 500 µL of 80 mM H_2_O_2_, and the decrease in absorbance was monitored at 240 nm for 1 min. CAT activity was calculated using the molar extinction coefficient (ε = 36 M^−1^ cm^−1^) and expressed as U mg^−1^ protein.

#### 4.4.3. Ascorbate Peroxidase (APX, EC 1.11.1.11) Activity

The reaction mixture consisted of 200 µL of extract, 300 µL of phosphate buffer (50 mM, pH 7.0), and 250 µL of 1 mM ascorbic acid. The reaction was initiated by adding 250 µL of 20 mM H_2_O_2_, and the decrease in absorbance was monitored at 290 nm for 1 min. APX activity was calculated using the molar extinction coefficient (ε = 2.8 mM^−1^ cm^−1^) and expressed as U mg^−1^ protein.

#### 4.4.4. Guaiacol Peroxidase (GOPX, EC 1.11.1.7) Activity

The reaction mixture consisted of 50 µL of extract, 500 µL of phosphate buffer (100 mM, pH 6.5), and 250 µL of 60 mM guaiacol. The reaction was initiated by the addition of 200 µL of 0.25% H_2_O_2_, and the increase in absorbance was monitored at 470 nm for 1 min. GOPX activity was calculated using the molar extinction coefficient (ε = 26.6 mM^−1^ cm^−1^) and expressed as U mg^−1^ protein.

#### 4.4.5. Glutathione Reductase (GR, EC 1.6.4.2) Activity

The reaction mixture consisted of 200 µL of extract, 300 µL of phosphate buffer (50 mM, pH 7.5), 100 µL of 35 mM MgCl_2_, and 100 µL of 5 mM L-oxidized glutathione. The reaction was initiated by the addition of 300 µL of 0.345 mM NADPH, and the decrease in absorbance was monitored at 340 nm for 1 min. GR activity was calculated using the molar extinction coefficient (ε = 6.2 mM^−1^ cm^−1^) and expressed as U mg^−1^ protein.

### 4.5. Element Concentrations in the Leaves

Dried leaf samples were ground into a fine powder and digested in concentrated HNO_3_ in Teflon PFA vessels using a microwave-assisted reaction system (MarsXpress, CEM Corp., Matthews, NC, USA). Element concentrations in the resulting digestates were determined by inductively coupled plasma mass spectrometry (ICP-MS; Agilent 7500CE, Santa Clara, CA, USA). To assess analytical precision and ensure quality control, NIST certified reference materials (1573a—tomato leaves and CTTA-OTL-1—oriental tobacco leaves), reagent blanks, and duplicates of every tenth sample were included.

### 4.6. Statistical Analysis

Each treatment consisted of three replicate plots per crop. The plot was considered the experimental unit, while three plants sampled within each plot for biochemical and elemental analyses, or 25 plants per plot for plant growth measurements, were treated as subsamples. Statistical analyses were performed using Statistica 14.0.0.15 (StatSoft, Cracow, Poland). To evaluate statistically significant differences between treatments, data for each species were analyzed separately using one-way ANOVA. Differences were considered significant at *p* < 0.05. Prior to the analyses, the assumptions of normality and homogeneity of variance were verified using the Shapiro–Wilk and Levene tests, respectively. Post hoc comparisons among means were performed using Fisher’s test. Heatmaps were generated in R software (version 2025.05.1+513) using the pheatmap (version 1.0.13) and corrplot (version 0.95) packages, based on standardized data. Dissimilarity between samples was calculated using Euclidean distance, followed by hierarchical clustering for data grouping. Principal component analysis (PCA) was conducted to obtain a holistic overview of the results and to evaluate independent trends in the variations among the investigated data, using three mean values representing three experimental plots per treatment. PCA was performed using MVSP software, version 3.1.

## 5. Conclusions

This study provides new insight into the physiological responses of bioenergy crops to chronic exposure to heavy metals under field conditions and into the role of biostimulants in modulating antioxidant defenses. Our results demonstrate that the biostimulant effects are strongly species-specific. Sorghum showed the most pronounced positive response, with improved growth and enhanced accumulation of non-enzymatic antioxidants, suggesting an important role of osmoprotective metabolites and active, coordinated adaptive response. In contrast, hemp responded primarily through activation of enzymatic antioxidant defenses, a pattern consistent with a potentially more costly stress response. Miscanthus maintained biomass despite oxidative signals and relatively limited physiological responses, indicating a stable tolerance-based strategy rather than a predominantly stress-responsive profile. These findings indicate that different plant species employ distinct physiological strategies to cope with metal-induced oxidative stress under the same environmental conditions.

Overall, the results provide a structured evaluation of the proposed hypotheses. The first hypothesis was partially supported, as humic substances improved plant growth and partially reduced oxidative stress in a highly species-specific manner, mainly in sorghum, while such effects were not evident in miscanthus. Importantly, the combined application of HSs and AMF did not result in a consistent synergistic effect on antioxidant pathways or stress-related metabolites, contrary to the second hypothesis. This suggests that interactions between different types of biostimulants and plant physiological processes are complex and depend on plant species, soil properties, and environmental conditions, highlighting the multifaceted nature of plant–biostimulant interactions. In contrast, the third hypothesis was confirmed, as all measured parameters revealed clear interspecific differences in physiological and biochemical responses. Our findings also indicate that oxidative stress-related parameters are highly species-specific and, therefore, cannot be considered universal biomarkers of plant tolerance to metal-polluted environments. These results emphasize the importance of considering species-specific strategies when selecting crops for cultivation on metal-polluted and other marginal lands.

Future research should investigate the underlying mechanisms of these differential responses by integrating physiological, biochemical, and molecular approaches. In particular, transcriptomic and metabolomic analyses would provide deeper insight into the regulatory networks governing plant responses to heavy metals and the mechanisms through which biostimulants modulate stress tolerance, thereby supporting the development of more effective phytomanagement strategies for contaminated environments.

## Figures and Tables

**Table 1:** Changes in growth parameters, stress-related metabolites, antioxidant enzyme activities, and leaf metal concentrations in hemp, sorghum, and miscanthus following application of biostimulants: humic substances (HS) and humic substances combined with mycorrhiza (HS + M). Colors indicate statistically significant differences relative to the control or between biostimulants: Light red—significantly higher than control, dark red—significantly higher than the control and the other biostimulant, light blue—significantly lower than control, lack of color—no significant difference relative to control.

Measured Parameter	Hemp		Sorghum		Miscanthus	
	HS	HS + M	HS	HS + M	HS	HS + M
Plant height						
Plant fresh weight						
TBARS						
Free proline						
Total sugars						
SOD activity						
CAT activity						
APX activity						
GOPX activity						
GR activity						
Zn concentration						
Cd concentration						
Pb concentration						

**Table 2:** Results of PCA based on the analyzed parameters in all three species—hemp, sorghum, and miscanthus collectively: (A) Eigenvalues and variance (%) explained by the first two PCA axes; (B) loading components for each variable associated with the two axes. For explanation of abbreviations see Figure 5.

Variables	Axis 1	Axis 2
(A)		
Eigenvalues	5.64	3.63
Percentage	43.36	27.93
Cum. percentage	43.36	71.29
(B)		
height	0.357	−0.182
FW	0.368	0.229
TBARS	−0.338	0.169
proline	0.228	−0.425
sugars	−0.352	−0.066
SOD	−0.339	0.019
CAT	0.295	−0.096
APX	0.124	−0.173
GOPX	−0.118	−0.451
GR	0.173	−0.217
Zn	0.223	0.332
Cd	0.369	0.203
Pb	0.005	0.508

**Table 3:** Results of PCA based on the analyzed parameters separately for hemp, sorghum, and miscanthus: (A) Eigenvalues and variance (%) explained by the first two PCA axes; (B) loading components for each variable associated with the two axes. For explanation of abbreviations see Figure 5.

	Hemp		Sorghum		Miscanthus	
Variables	Axis 1	Axis 2	Axis 1	Axis 2	Axis 1	Axis 2
(A)						
Eigenvalues	7.65	2.56	6.63	2.73	5.02	3.18
Percentage	58.85	19.69	50.97	21.00	38.65	24.45
Cum. percentage	58.85	78.54	50.97	71.97	38.65	63.10
(B)						
height	0.275	0.014	0.272	0.004	0.251	0.187
FW	0.234	0.3	0.281	−0.327	−0.033	−0.103
TBARS	−0.12	0.562	−0.361	−0.192	0.417	0.032
proline	−0.315	0.218	0.365	−0.061	0.036	0.549
sugars	0.246	−0.005	0.315	−0.293	−0.364	−0.286
SOD	−0.265	0.025	−0.031	0.483	−0.169	0.116
CAT	0.342	0.079	−0.035	0.348	−0.379	−0.027
APX	−0.34	0.066	0.186	0.3	0.2	−0.178
GOPX	−0.214	0.437	0.011	0.4	−0.174	0.428
GR	−0.05	−0.501	0.196	−0.3	0.138	0.003
Zn	−0.327	−0.245	0.355	0.218	−0.42	0.166
Cd	−0.349	0.023	0.375	0.121	−0.277	0.431
Pb	−0.34	−0.18	0.377	0.103	−0.338	−0.357

## Data Availability

The original contributions presented in this study are included in the article and Supplementary Materials (Table S1). Further inquiries can be directed to the corresponding author.

## Supplementary Materials

The following supporting information can be downloaded at: https://www.mdpi.com/article/10.3390/ijms27093942/s1.
