# Supplementary material for: Species-Specific Effects of Humic Substances and Mycorrhiza on Antioxidant Defense and Metal Stress Tolerance in Cannabis sativa, Sorghum sudanense × bicolor, and Miscanthus × giganteus Under Field Conditions
Source: Int J Mol Sci. 2026 Apr 28;27(9):3942. doi: 10.3390/ijms27093942 (PMC13163904; doi:10.3390/ijms27093942)
Supplement: Supplementary file 1 [file ijms-27-03942-s001.zip › ijms-4239639-supplementary.pdf]

Table S1. Growth parameters (shoot fresh weight and plant height), concentrations of stress-related markers (TBARS, proline, and total sugars), activities of antioxidative enzymes (SOD, CAT, APX, GOPX, and GR), and concentrations of zinc (Zn), cadmium (Cd), and lead (Pb) in the leaves of hemp, sorghum, and miscanthus grown on metal-contaminated soil without biostimulant application (C – control) or with humic substances (HS) or humic substances combined with mycorrhiza (HS+M). Data are presented as means ± SD; the *n* value is provided for each parameter. Abbreviations: TBARS, thiobarbituric acid reactive substances; SOD, superoxide dismutase; CAT, catalase; APX, ascorbate peroxidase; GOPX, guaiacol peroxidase; GR, glutathione reductase.

**Analyzed parameter**	**Treatment**	**Hemp**	**Sorghum**	**Miscanthus**
fresh weight [g plant^{-1}], n=9	C	0.259 ± 0.049	0.569 ± 0.055	2.624 ± 0.420
	HS	0.235 ± 0.041	0.717 ± 0.074	2.964 ± 0.414
	HS+M	0.241 ± 0.048	0.726 ± 0.110	3.098 ± 0.397
height [m], n=75	C	2.930 ± 0.476	3.652 ± 0.218	3.331 ± 0.121
	HS	2.903 ± 0.280	3.865 ± 0.272	3.396 ± 0.168
	HS+M	2.860 ± 0.316	3.750 ± 0.265	3.349 ± 0.364
TBARS [µmol g FW^{-1}], n=9	C	7.056 ± 0.414	4.823 ± 0.675	3.653 ± 0.792
	HS	7.000 ± 0.837	2.194 ± 0.562	5.011 ± 1.231
	HS+M	7.033 ± 0.660	4.274 ± 0.647	5.839 ± 0.715
proline [µmol g^{-1} FW], n=9	C	243.01 ± 19.51	874.87 ± 102.19	1348.33 ± 204.23
	HS	257.04 ± 18.45	1394.14 ± 86.66	1081.94 ± 143.36
	HS+M	283.48 ± 24.77	1115.26 ± 111.77	1528.33 ± 167.04
total sugars [µg mg^{-1} FW], n=9	C	792.93 ± 175.75	162.97 ± 29.07	747.21 ± 50.63
	HS	760.50 ± 154.88	293.83 ± 50.91	709.24 ± 126.76
	HS+M	563.88 ± 124.11	285.27 ± 59.24	604.19 ± 125.49
SOD [U mg^{-1} protein], n=9	C	187.03 ± 22.06	141.91 ± 17.80	215.25 ± 36.09
	HS	235.47 ± 46.88	124.96 ± 9.33	178.54 ± 37.20
	HS+M	266.64 ± 55.21	77.52 ± 8.28	196.31 ± 39.96
CAT [U mg^{-1} protein], n=9	C	0.805 ± 0.147	1.189 ± 0.364	1.075 ± 0.256
	HS	0.413 ± 0.132	1.194 ± 0.371	0.690 ± 0.288
	HS+M	0.326 ± 0.126	0.827 ± 0.424	0.518 ± 0.193
APX [U mg^{-1} protein], n=9	C	0.004 ± 0.002	0.015 ± 0.005	0.010 ± 0.005
	HS	0.010 ± 0.003	0.019 ± 0.007	0.017 ± 0.007
	HS+M	0.015 ± 0.003	0.011 ± 0.003	0.015 ± 0.006
GOPX [U mg^{-1} protein], n=9	C	0.083 ± 0.016	0.102 ± 0.024	0.477 ± 0.103
	HS	0.073 ± 0.013	0.103 ± 0.022	0.246 ± 0.061
	HS+M	0.102 ± 0.015	0.083 ± 0.023	0.441 ± 0.080
GR [U mg^{-1} protein], n=9	C	0.0016 ± 0.0003	0.0018 ± 0.0006	0.0022 ± 0.0006
	HS	0.0021 ± 0.0005	0.0027 ± 0.0008	0.0025 ± 0.0004
	HS+M	0.0017 ± 0.0004	0.0030 ± 0.0007	0.0025 ± 0.0008
Zn [mg kg^{-1} DW], n=3	C	129.423 ± 12.02	158.477 ± 22.04	158.810 ± 15.19
	HS	168.171 ± 38.02	269.861 ± 35.74	65.044 ± 13.81
	HS+M	175.554 ± 42.17	167.193 ± 12.77	70.295 ± 23.97
Cd [mg kg^{-1} DW], n=3	C	1.021 ± 0.313	4.394 ± 0.854	1.142 ± 0.322
	HS	1.554 ± 0.218	6.765 ± 0.636	0.728 ± 0.079
	HS+M	2.507 ± 0.561	4.978 ± 0.811	0.975 ± 0.453
Pb [mg kg^{-1} DW], n=3	C	31.126 ± 2.32	13.077 ± 2.96	3.245 ± 2.77
	HS	40.517 ± 4.65	24.254 ± 3.17	4.648 ± 3.46
	HS+M	44.471 ± 3.29	15.893 ± 6.11	3.538 ± 2.24
